# Post-translational Modification Crosstalk and Hotspots in Sirtuin Interactors Implicated in Cardiovascular Diseases

**DOI:** 10.3389/fgene.2020.00356

**Published:** 2020-04-30

**Authors:** Suruchi Aggarwal, Sanjay K. Banerjee, Narayan Chandra Talukdar, Amit Kumar Yadav

**Affiliations:** ^1^Translational Health Science and Technology Institute, NCR Biotech Science Cluster, Faridabad, India; ^2^Division of Life Sciences, Institute of Advanced Study in Science and Technology, Guwahati, India; ^3^Department of Molecular Biology and Biotechnology, Cotton University, Guwahati, India

**Keywords:** cardiovascular diseases, hotspot, cancer, protein–protein interactions, neurodegenerative, modifications, crosstalk, variant

## Abstract

Sirtuins are protein deacetylases that play a protective role in cardiovascular diseases (CVDs), as well as many other diseases. Absence of sirtuins can lead to hyperacetylation of both nuclear and mitochondrial proteins leading to metabolic dysregulation. The protein post-translational modifications (PTMs) are known to crosstalk among each other to bring about complex phenotypic outcomes. Various PTM types such as acetylation, ubiquitination, and phosphorylation, and so on, drive transcriptional regulation and metabolism, but such crosstalks are poorly understood. We integrated protein–protein interactions (PPI) and PTMs from several databases to integrate information on 1,251 sirtuin-interacting proteins, of which 544 are associated with cardiac diseases. Based on the ∼100,000 PTM sites obtained for sirtuin interactors, we observed that the frequency of PTM sites (83 per protein), as well as PTM types (five per protein), is higher than the global average for human proteome. We found that ∼60–70% PTM sites fall into ordered regions. Approximately 83% of the sirtuin interactors contained at least one competitive crosstalk (*in situ*) site, with half of the sites occurring in CVD-associated proteins. A large proportion of identified crosstalk sites were observed for acetylation and ubiquitination competition. We identified 614 proteins containing PTM hotspots (≥5 PTM sites) and 133 proteins containing crosstalk hotspots (≥3 crosstalk sites). We observed that a large proportion of disease-associated sequence variants were found in PTM motifs of CVD proteins. We identified seven proteins (TP53, LMNA, MAPT, ATP2A2, NCL, APEX1, and HIST1H3A) containing disease-associated variants in PTM and crosstalk hotspots. This is the first comprehensive bioinformatics analysis on sirtuin interactors with respect to PTMs and their crosstalks. This study forms a platform for generating interesting hypotheses that can be tested for a deeper mechanistic understanding gained or derived from big-data analytics.

## Introduction

Sirtuins are a class of lysine deacetylases that remove acetylation from important metabolic proteins and regulate diverse metabolic processes. Sirtuins are class III histone deacetylases (HDACs) that use NAD+ as a cofactor for their activity. The other classes (I, II, and IV) use zinc ion for the enzymatic activity (Seto and Yoshida, 2014). During calorie restriction or exercise condition, sirtuins show enhanced activity compared to HDACs because of their dependence on NAD+ for enzymatic activity. This makes them an important player in all major metabolic processes. There are seven sirtuins in the human sirtuin family, three of which are primarily found in the nucleus (SIRT-1, SIRT-6, and SIRT-7), other three in the mitochondria (SIRT-3, SIRT-4, and SIRT-5), whereas SIRT2 is found primarily in the cytoplasm (Masoro, 2004; Milne and Denu, 2008). All of them are known to play important roles in various diseases such as cancers, Alzheimer disease, insulin resistance, aging, diabetes, and inflammation. Their roles in cardiovascular diseases (CVDs) have also been interrogated extensively, and further exploration of sirtuins is necessary to aid in development of therapeutics for cardioprotection (Porter et al., 2012; Gajjala et al., 2015; Sun et al., 2018).

Sirtuins not only deacetylate proteins but also play a role in removal of other acylations such as malonylation, succinylation, crotonylation, butyrylation, and so on (Olsen, 2014). Acylations are added mostly via a non-enzymatic mechanism, but some acyl transferases (lysine acetyl/acyl transferases, also called KATs) are known to acylate major regulatory proteins (Chen et al., 2007; Sabari et al., 2017). Some of the acylations, such as acetylation, are known to stabilize a protein that is otherwise condemned for degradation by ubiquitination. Acetylations have been historically known to be associated with chromatin regulation via modification of the histone tails. Many epigenetic studies have revealed what is labeled as the “histone code,” depicting an intricate crosstalk of acetylation, methylation, and ubiquitination on specific histone lysine sites for deactivating or activating DNA transcription (Jenuwein and Allis, 2001; Schwammle et al., 2016). Crosstalks between different acylations, such as acetylation, butyrylation, malonylation, succinylation, and so on, have also been highlighted on numerous occasions (Olsen, 2014; Sabari et al., 2017). Negative crosstalks at the same site between various lysine modifications (referred to as *in situ* crosstalks from here on) have been observed quite frequently. Acetylation and ubiquitination *in situ* crosstalks have been highlighted in several large-scale proteome studies (Minguez et al., 2012), but the effect of their subsequent interactions and effect on other lysine modifications are still underexplored.

While sirtuins have been proposed as a therapeutic target for various cancers, aging, and neurodegenerative disorders, they have also been explored as potential targets for CVDs (Masoro, 2004; Yamamoto et al., 2007; Milne and Denu, 2008). Their role in cardioprotection has been extensively explored in context of the flipping post-translational modification (PTM) switches to enhance or to diminish any protein function. In rats and mice, enhancing or maintaining the normal sirtuin activity has shown a beneficial effect in cardiac diseases (Porter et al., 2012). Post-translational modifications (PTMs) and their interplays not only regulate the enzymatic switches but also decide the cellular localization and protein–protein interaction (PPI)–mediated functions of many cardioprotective proteins (Lin and Begley, 2011). This interplay can also lead to a “loss of function” or “gain of function” in regulating protein activity (Yang and Seto, 2008).

Studying the interactors of sirtuins can help in better understanding of their potential roles and effects of PTMs in these proteins. The sirtuin interactors have been earlier explored to describe network level properties for the interactions in great detail (Sharma et al., 2013). However, their other important properties such as structural disordered regions, role in diseases, PTM sites, and types have not been well characterized. In order to study the various PTM-related properties of sirtuin interactors, we provide a detailed bioinformatics analysis on sirtuin interactors and their various properties integrating large-scale data from diverse sources. We have curated a dataset of sirtuin interactors from several databases to study their PTM properties such as PTM hotspots and PTM crosstalk in context of known disease variants. To the best of our knowledge, this is the first study that deals with PTMs and their crosstalk in sirtuin interactors. The deeper understanding can aid in better mechanistic hypothesis generation and selection of diagnostic/therapeutic targets for future studies based on these properties.

## Materials and Methods

### Integration of Protein–Protein Interactions for Sirtuins

Data from five databases, APID (Alonso-Lopez et al., 2016), BioGRID (Chatr-Aryamontri et al., 2017), HIPPIE (Alanis-Lobato et al., 2017), STRING (Szklarczyk et al., 2015, 2017), and IntAct (Hermjakob et al., 2004; Kerrien et al., 2012), were downloaded. The databases were parsed using in-house Perl scripts. The proteins common to any two databases were selected for further study (Supplementary Figure S1). Of a total 23,266 proteins, 1,258 proteins were selected for their direct interactions with sirtuins. These 1,258 proteins were queried back in each database to identify the first-order interactions between sirtuins and among themselves. From these interactions, total degree was calculated for each protein for first-order interactions (85,381), and 75th percentile from the maximum was used as the criterion to describe a particular protein to be a hub protein. STRING database server was used for PPI analysis of specific subsets of proteins identified in this study.

### Integration of Post-translational Modification Sites

Three databases, dbPTM (Lee et al., 2006; Huang et al., 2019), PhosphositePlus (PSP) (Hornbeck et al., 2012, 2015), and neXtprot (Lane et al., 2012), were used to get the information of modification data. The process for each of these has been described in the following sections.

#### dbPTM

dbPTM contains a collection of 33 types of PTMs. The data were downloaded from http://dbptm.mbc.nctu.edu.tw/download.php. The downloaded files were processed using in-house Perl scripts, and data pertaining to human PTM sites were filtered for further analysis. There were 438,463 modified sites reported in humans in dbPTM.

#### PhosphositePlus

PhosphositePlus contains a collection of seven types of PTMs. The data were downloaded from https://www.phosphosite.org/staticDownloads. The files were combined and filtered for PTM sites present only in humans. The total modified sites obtained from PSP were 387,432.

#### neXtProt

neXtProt is a protein database containing information about human proteins, and their properties manually curated from literature by expert curators. It contains information about amino acid modifications, crosslinks, glycosylation, and lipidation sites in human proteins. The XML file containing all data was downloaded from ftp://ftp.nextprot.org/pub/current_release/and was programmatically parsed to obtain information about PTM sites. There were 188,851 sites reported in neXtProt.

Once the data were obtained and parsed from the three databases, it was programmatically combined, and redundancy in PTM sites was removed. The PTM names were manually checked, and common names were added to the PTMs to allow for easy analysis of PTM types. For example, phosphoserine, phosphothreonine, and phosphotyrosine were collectively called phosphorylation. Only the data corresponding to sirtuin interactors were kept for further analysis, which contained 89,111 sites.

### Disease Classification

The list of disease–gene association was downloaded from DisGeNET (Pinero et al., 2015) database and was parsed using an in-house script into a manageable tabular format. The list of diseases was manually curated to identify CVDs separately from the rest of the diseases. The list of proteins interacting with SIRTs (1,258) was queried in this curated table, and the proteins were segregated in three categories: proteins associated with CVDs, proteins associated with other diseases (not CVDs), and proteins not known to be associated with any disease. Hereafter, these categories are represented as CVD, OD, and ND, respectively, for the sake of brevity. The proteins in CVD category may also be associated with other diseases as well.

### Disorder Prediction

For the sirtuin interactor proteins containing PTMs (1,251 proteins), sequences were downloaded from neXtProt database. Structural disorder for these proteins was predicted using the standalone version of DISOPRED3 (Jones and Cozzetto, 2015) and fMoRFpred webserver^1^ (Yan et al., 2016). The fMoRFpred server uses IUpred (Dosztanyi et al., 2005), PSIPRED (Jones, 1999), and Espritz (Walsh et al., 2012) software for disorder prediction. The data were segregated into three categories: ordered, disordered-binding, and disordered non-binding as per the outputs. An in-house Perl program was used to identify PTM positions in the three types of structural regions as identified by DISOPRED3 and fMoRFpred.

### Gene Ontology and Pathway Analysis

The SIRT interactors list (1,258 genes) was used as input for Gene Ontology (GO) analysis using the PANTHER (Mi et al., 2019) classification system^2^ to fetch the annotations in the three GO categories: biological process, molecular function, and cellular component. It was also used to fetch protein classes. STRING v11 database webserver^3^ (Szklarczyk et al., 2019) was queried with high confidence threshold of 0.7, used for KEGG and Reactome pathway analysis at a false discovery rate threshold of ≤0.05.

### PTM Crosstalk

For *in situ* crosstalk, that is, competition of PTMs on the same site, the data combined from the three sites (dbPTM, neXtProt, and PSP) were used to identify different PTMs on the same amino acid position. About 11,400 PTM sites were identified containing *in situ* crosstalk.

For finding *cis*-crosstalk, data were downloaded from PTMcode (Minguez et al., 2013) database^4^. The PTM sites occurring in the 1,251 sirtuin interactors with residue conservation score of ≥ 80 were used for further analysis.

### Hotspot Analysis

For all the PTM sites, a motif of ±7 amino acid stretch was extracted from the corresponding protein sequence. For each PTM site, the frequency of other co-occurring PTM sites was calculated in the vicinity for the defined motif. Every motif that contained ≥5 PTM sites excluding the central PTM was called as *PTM hotspot region*. If a motif contained ≥3 sites harboring more than two PTMs competing for the same site (i.e., ≥3 *in situ* crosstalks), it was defined as a *(PTM) crosstalk hotspot*. It is possible to have overlapping regions with hotspots for both PTM and crosstalks. Such regions were stitched together to calculate an overall actual length covered. It is possible for a protein to lack a PTM hotspot but contain a crosstalk hotspot.

### Variant Identification

To identify variants within the PTM motif, data were downloaded from Humsavar (The UniProt, 2017)^5^ and PTMvar (Hornbeck et al., 2015)^6^. The data were programmatically parsed using Perl script, and variants at PTM sites were mapped for the sirtuin interactors. As before, the variants were mapped onto the motif of ±7 amino acids around a PTM site. Any variant overlapping exactly at the site of modification or in vicinity (within the motif) around it was considered for further analysis.

## Results

**FIGURE 1:**
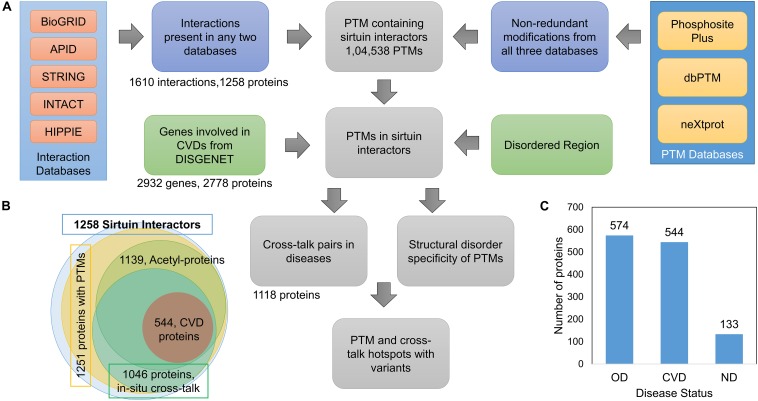
**(A)** Schematic overview of workflow to extract sirtuin interactors and their PTM sites from different databases. **(B)** Overview of proteins involved in interactions with SIRTs, the interactors containing PTMs, acetyl-containing proteins, and proteins with *in situ* crosstalks. It also shows proteins known to be associated with cardiovascular diseases (CVDs). **(C)** Proteins in the SIRTUIN interaction network to be associated with CVDs, other diseases (OD), and no-disease (ND).

We downloaded the data from five different PPI databases, namely, BioGRID, APID, IntAct, HIPPIE, and STRING. After integrating the SIRT interactors, we got 1,258 proteins in total with 1,610 direct interactions with one or more of the seven sirtuins (Figure 1A).

We first describe the distribution of PTM types and sites in the sirtuin interactors. We describe how different PTMs are distributed among these interactors to study the trends of important PTMs and their crosstalks. Easy availability of data from large-scale studies in these databases of PTM sites makes it amenable for studying such patterns in specific subset of proteins. While this study from integrated large-scale curated data may suffer from the biases of studies on the popular PTMs (such as phosphorylation, acetylation, methylation, ubiquitination, etc.), the associations are still meaningful and may be enriched further when more data are available on lesser-studied PTM types. Some of the PTMs have been studied extensively because of advances in enrichment methods, high abundance, or their functional importance and popularity. It may also reflect their association with the diseases. As the interactions and modification sites are compiled from a vast corpus of literature by several databases, this is a highly useful data resource to study PTMs and their crosstalk in association with sirtuin-interacting proteins. All seven sirtuins have been studied as therapeutic target in CVDs (Sebastian et al., 2012; Adam et al., 2013; Yi et al., 2017; D’Onofrio et al., 2018; Sun et al., 2018; Donniacuo et al., 2019). However, their interactors have not been explored extensively with respect to their PTMs and their crosstalks, or finding regions with extensive hotspots for such sites, which are generally considered to have higher importance for increased functional repertoire of proteins or involvement in diseases. It has already been shown that sites of multiple PTMs are more prone to be involved in diseases, and more details for specific proteins will help in deeper mechanistic understanding. Highly connected nodes in a network also depend on multiple functionalities by the interactions of proteins mediated by their PTMs. Finding these proteins will help in deducing mechanistic links into diseases and targets for further studies. With this study, we aim to highlight the various properties of sirtuin interactors with respect to their structural disorder, PTMs, and their crosstalk, variants, and search for PTM and crosstalk hotspots that can find important modulators of cellular metabolism. A deeper understanding can guide better therapeutic strategies for cardiovascular and metabolic disorders.

### Sirtuin Interactors and Diseases

After integrating data from five interaction databases (Figure 1A), 1,258 proteins were observed to be interacting with at least one SIRT, of which 1,251 proteins have at least one PTM site (Figure 1B and Supplementary Table S1). As the sirtuins remove the acetyl group from the substrates, most interactors are expected to have acetylation or other PTMs for interactions. We focused on interactors with at least one PTM site of any PTM type. Although there were only 1,135 proteins containing one or more acetylation, all PTM-containing proteins were used for further analysis (Figure 1B). There were 356 proteins interacting with more than one SIRT (Supplementary Table S1). These proteins included 56 transcription factors such as FOXOs, TP53, MYC, and so on. Although all seven sirtuins have been reported to play a crucial role in a variety of diseases, these have been specifically reported to be important in CVDs and aging (Law et al., 2009; Sebastian et al., 2012; Adam et al., 2013; Winnik et al., 2015; Grabowska et al., 2017; Yi et al., 2017; Carrico et al., 2018; D’Onofrio et al., 2018; Sun et al., 2018; Barjaktarovic et al., 2019; Donniacuo et al., 2019; Gomes et al., 2019). The 1,251 proteins were queried against DisGeNET database to fetch their disease associations, of which 1,118 proteins were mapped to diseases. Of these, 544 proteins mapped to CVDs; 574 proteins to other diseases (OD), such as cancer, Alzheimer disease, Parkinson disease, and so on; and 133 proteins did not map to any known disease, labeled as no disease (ND) (Figure 1C). We also checked if proteins involved in CVDs are also involved in some other diseases. We found that only four proteins out of 544 are unique to CVD (MYEF2, RBM25, TSR1, GNL2), and the rest were also involved in other diseases.

### PTM Distribution in the SIRT Interactors

**FIGURE 2:**
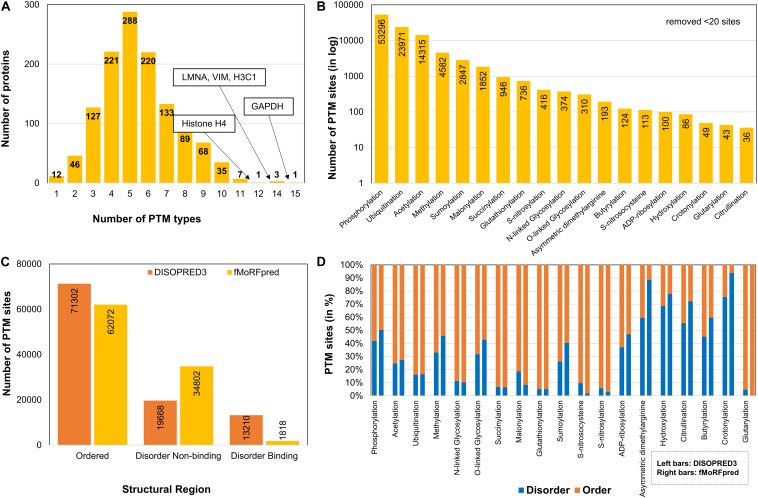
Dataset description after parsing and integrating for different PTM types in sirtuin interactors. **(A)** Frequency bar chart depicting the number of proteins that contain one or more PTM types. Most histones and GAPDH contained a higher number of PTM types than other proteins. **(B)** Distribution of sites for each PTM type shows phosphorylation, ubiquitination, and acetylation to be most predominant ones in sirtuin interactors. **(C)** Post-translational modification sites found in structural regions as predicted by DISOPRED3 and fMoRFpred. It shows most PTM sites map to ordered regions. **(D)** The division of each PTM in the type of structural region.

Post-translational modification sites were integrated from three databases – dbTPM, PSP, and neXtProt (Figure 1A). After the redundant sites with the same modification type at the same site were removed, the data contained 104,538 PTMs at 89,111 amino acid sites corresponding to 51 PTM types. On an average, 83 PTM sites per protein were observed, which is much higher than the global average of 4.5 residues per protein as reported by Minguez et al. (2012). The distribution of the number of PTM types and the number of proteins containing them are shown in Figure 2A. We observed that some proteins (GAPDH, LMNA, VIM, H3C1, and H4C1) harbor more than 10 PTM types. On an average, the sirtuin interactors have about five PTM types (Figure 2A), whereas the human proteome contains about two PTM types per protein as per data collected by us. As expected, most of the sites are dominated by phosphorylation, ubiquitination, and acetylation (Figure 2B) owing to availability of high-throughput methods among other factors. We also compared the number of PTM types in the different disease classes (CVD, OD, or ND) of the proteins, and we observed that the PTM distribution is similar in CVD-specific and other disease proteins (Supplementary Figure S2). This hints toward the predominant role of SIRTs and their interactors in regulating diverse cellular processes and maintaining the cellular harmony. Dysregulation of modifications by inactivation of SIRTs may change the cellular functional network leading to metabolic and cellular disarray causing many diseases.

### PTM and Protein Structure

Many studies have reported that PTMs mostly prefer disordered regions than ordered regions (Iakoucheva et al., 2004; Xie et al., 2007). The presence of a PTM in disordered region can cause disorder-to-order transition, thereby affecting a protein’s function or its interactions. This may help in diversifying the functional roles of the protein through creation of structurally new sites or PPI by providing a binding interface. Previously, many studies have enumerated the structural preference of popular modifications such as phosphorylation over an evolutionary scale either among a genus (Studer et al., 2016) or among kingdoms (Ward et al., 2004; Darling and Uversky, 2018). Interestingly, when we compared the PTM sites in structural regions, we found that ∼60–70% of the sites fall in ordered regions and the rest in disorder regions. Although the two tools used for disorder prediction (DISOPRED3 and fMoRFpred) do not agree on disorder-binding and disorder non-binding regions (Figure 2C), the observation about percentage disorder remains similar (Supplementary Figure S3). Even for phosphorylation, approximately half (50–60%) of modified sites identified in sirtuin interactors was present in ordered regions (Figure 2D). Other PTMs, such as ubiquitination, N-glycosylation, acetylation, and few other acylations (such as malonylation, succinylation, and glutarylation), are mostly in ordered regions (>70%). While phosphorylation is usually reported to have disorder-region preference, acetylation, ubiquitination, and methylation have been previously observed mostly in ordered regions (Narasumani and Harrison, 2018). Butyrylation (∼50%) and crotonylation (>70%) are acylations that contain more predicted disorder sites. Our data suggest some deviation from this trend for phosphorylation, whereas other observations corroborated well with previous literature reports.

### GO Analysis of SIRT Interactors

Gene enrichment analysis of the SIRT interactors revealed important cellular regulatory functions important for metabolism. Important categories in GO-Biological Processes include membrane enclosed lumen, cellular organelles, protein containing complexes, supramolecular complex, and so on. GO–Molecular Function category includes binding and catalytic activity, transcriptional regulation, structural molecule activity, transportation, and so on. GO–Cellular Component category includes biological regulation, cellular component organization or biogenesis, cellular processes, localization, response to stimulus, signaling, and metabolic processes (Supplementary Figures S4A–C). We  also analyzed the PANTHER protein classes, which revealed intercellular signal molecules, calcium binding, cell adhesion, chromatin regulation, cytoskeletal proteins, transcription regulators, membrane trafficking proteins, protein modifying enzymes, scaffold proteins, protein binding regulators, translation proteins, and transporters. As expected, most of these processes and categories of proteins reflect the central role sirtuins play in metabolism and maintaining cellular processes (Supplementary Figure S4D). The protein classes depict that, in addition to broad metabolic functions, these proteins also play a regulatory role in metabolic processes important for cardiac function.

These proteins were queried using STRING v11 to fetch KEGG and Reactome pathways. KEGG pathways revealed ribosome biogenesis, RNA transport and metabolism, central carbon metabolism, pyruvate metabolism, and various pathways in metabolic diseases and cancers. Reactome pathways also revealed similar terms such as RNA metabolism, cell cycle, gene expression, translation, PTMs, metabolism, chromatin regulation, signal transduction, and so on (Supplementary Data Sheet 1). These pathways suggest a broad role of these proteins in regulating important metabolic processes.

### PTM Crosstalk in SIRTUIN Interactors

Post-translational modifications mediate the rapid responses to external stimuli by signaling. The signaling aberrations can cause dampened or heightened PTM signals and have been described well in literature (Liddy et al., 2013; Ardito et al., 2017). However, how PTMs crosstalk by cooperativity or competition has not been explored for sirtuin interactors in particular. The PTMs and their crosstalk constitute a biological regulatory layer that has evaded much insight until recently as it does not require genomic regulation or quantitative changes in protein levels. The regulation of protein function via competitive sites has been extensively explored for some proteins such as p53 where the extensive crosstalks of acetylation, methylation, and ubiquitination govern DNA damage response (Yang and Seto, 2008; Fan et al., 2014). The kinase reprogramming via multiple phosphorylation sites in MAPK and JNK signaling pathways has also demonstrated an alternative pathway of drug resistance in cancer (Aggarwal et al., 2017). This makes it necessary to understand the crosstalk of multiple PTMs.

**FIGURE 3:**
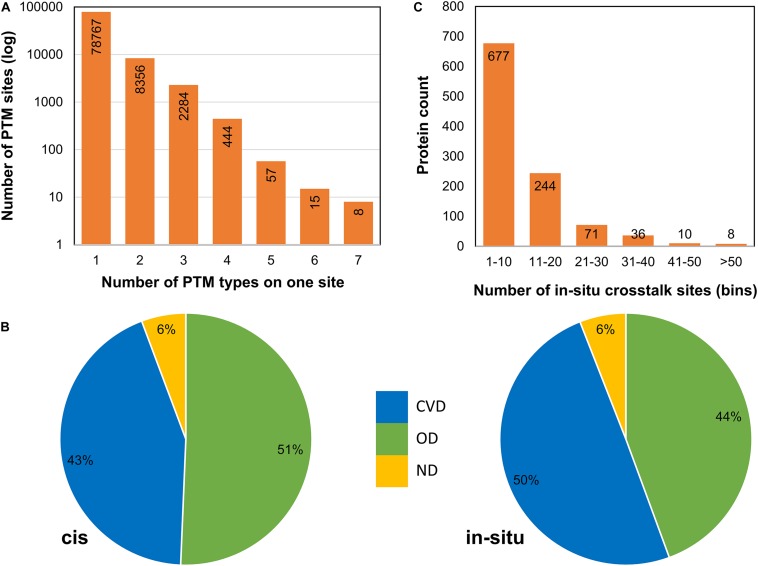
Post-translational modification crosstalks in sirtuin interactor proteins. **(A)** The frequency of proteins with number of PTM types at each site. Apart from the first bar (with single PTM type), the rest have *in situ* crosstalks. **(B)** *cis* and *in situ* crosstalks for sirtuin interactors reveal that most crosstalks occur in CVDs and other diseases, whereas very few (only 6% sites) were not involved with any disease. The *in situ* crosstalk seems to play an important role in CVDs in sirtuin interactors. **(C)** The frequency of proteins in each bin of *in situ* crosstalk sites reveals that most proteins have 1–10 crosstalk sites. Few proteins have more than 50 such sites.

To analyze PTM crosstalk, we downloaded data for PTM coevolution and crosstalk (*cis*-PTM crosstalk) from PTMcode database. We also identified *in situ* crosstalks (PTM competition on same site) from the data collated from the three databases. For each PTM site, we counted the frequency of competing PTM types at that position, where one denotes only one PTM type (meaning no competition), two means there are two types of PTMs (such as acetylation and ubiquitination competing for the same site), and so on (Figure 3A). We observed most PTM sites to be occurring with a single PTM type while a sizeable fraction (∼11,164 sites of total 89,111 PTM sites) to contain competing modification types. This denotes that there is extensive negative PTM crosstalk data available in literature hiding in plain sight. These data can be studied to probe deeper into crosstalk mechanisms and glean novel mechanistic or biological understanding of sirtuin interactors. We also mapped sirtuin interactors and DisGeNET disease associations to find if there is a propensity of crosstalk in diseases for sirtuin interactors. Here, we mapped both *cis* and *in situ* crosstalk data for disease associations. We observed that both types of PTM crosstalk are found aplenty in most of the disease-related proteins (Figure 3B). We then asked how the different types of PTM crosstalks are distributed in the interactors and types of diseases. Cardiovascular disease proteins have more *in situ* crosstalks but fewer *cis* crosstalks than OD, even though numbers are not too different (Figure 3B). This suggests a differential mechanism of PTM crosstalk roles in different types of diseases where *in situ* crosstalk may be driving differential interactions in CVDs. However, we only proceeded for in-depth analysis of *in situ* crosstalks. As a study of *cis*-crosstalk is more prediction based, we kept it outside the purview of this study.

For *in situ* crosstalk, there are many examples already reported in literature such as acetylation–methylation (Ac-Me) and acetylation–ubiquitination (Ac-Ub) crosstalk. We studied the occurrence of multiple crosstalk sites in sirtuin-interacting proteins. We found that of the 1,251 proteins, 1,046 proteins (∼83%) have at least one site that involves a PTM crosstalk (Figure 3C). While most of the proteins carry ≤10 *in situ* crosstalk sites, there were 367 proteins that harbored more than 10 sites of *in situ* crosstalk. For example, AHNAK, a calcium transporter with an important function in cardiac regulation, harbors 339 PTM crosstalk sites, highest among all sirtuin interactors.

In previous studies, it has been reported that *in situ* PTM sites have a higher tendency to occur in disordered regions (Pejaver et al., 2014). Surprisingly, we observed that most of the crosstalk PTM sites were observed in predicted ordered regions (Supplementary Figure S5) by DISOPRED3, as well as fMoRFpred. Because these sites are found in sirtuin interactors, their crosstalk in ordered regions represents their importance on an evolutionary scale.

On further investigation, we found that of ∼87,000 PTM sites, ∼11,000 sites were found to harbor PTM crosstalks (Figure 3A). Most of these sites are observed to contain competitive crosstalk between two PTMs (e.g., Ac-Ub occurring on ∼5,000 sites). The predominant crosstalks found were mostly of different acylations (i.e., acetylation. succinylation, malonylation, glutarylation, butyrylation, crotonylation, etc.) vs. either of ubiquitination or sumoylation, but sometimes also methylation (Supplementary Figure S6). The sites with higher number of competing PTMs (six and seven PTMs per site) are all reported in histones as expected, consistent with histone PTM code reports. Vimentin was a notable exception. In histones, crosstalks among methylation (mono, di, and tri) and acetylation on histone sites H3K4, H3K9, H3K27, and H3K36 are known to control transcriptional activity on the genomic regions (Jenuwein and Allis, 2001). Vimentin is a structural protein responsible for cell adhesion and migration. It is known for epithelial-to-mesenchymal transition in cancers and inflammatory reactions in atherosclerosis. It is a marker for coronary artery disease, and its methylated form is known to be a biomarker for colon cancer (Ulirsch et al., 2013; Gong et al., 2019).

**FIGURE 4:**
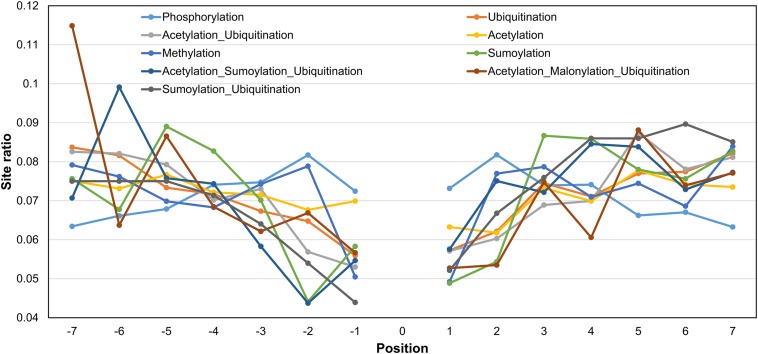
Fraction of sites around the central position for various PTMs and their *in situ* crosstalks. The *x*-axis represents the PTM motif position, and *y*-axis represents the ratio of sites present on that position with respect to the total number of sites for a particular PTM/crosstalk excluding the central site. While phosphorylation tends to decrease with distance from center, acetylation, ubiquitination, and their combination follow opposite trend. The competition between acetylation–malonylation and ubiquitination shows a preference for -7 position, whereas competition between acetylation, sumoylation, and ubiquitination shows preference for -6 position in the motif.

**FIGURE 5:**
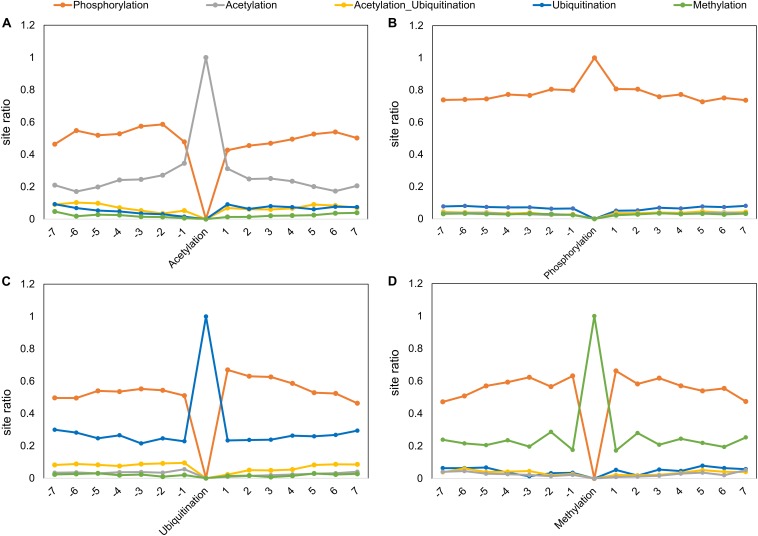
Post-translational modification motifs and occurrence of other PTMs or combination around **(A)** Acetylation, **(B)** phosphorylation, **(C)** ubiquitination, and **(D)** methylation. The *x*-axis represents the site position in the motif. The *y*-axis represents the ratio of sites with respect to the total sites for a particular PTM.

We also studied the distribution of a PTM (or crosstalk) site observed at different positions in PTM motifs (within ±7 amino acids, central site frequency not considered). Even though the overall observation of identifying other PTMs closer to the central PTM is low, some types of modifications have a higher tendency of occurrence in the vicinity (Figure 4). For example, a competitive crosstalk of lysine modification containing acetylation, malonylation, or ubiquitination has the highest probability of occurring at the -7 position among all the sites observed for this combination (Figure 4). Another crosstalk (acetylation, sumoylation, and ubiquitination) was observed with greater occurrence at -6, -4, and -5 positions. This may be due to the higher density of crosstalk sites occurring in the same regions. For ubiquitination and acetylation without crosstalk, the propensity of same modification occurring in the motif reduces with the distance from the central site. Contrary to this, phosphorylation and methylation have a higher propensity of modification closer to the central site as opposed to farther from the center (Figure 4). However, this analysis does not provide information about individual PTMs and their relationships with respect to other PTMs. When we separately plot the fractional PTM sites observed for several top PTMs (including the central site) such as acetylation, phosphorylation, ubiquitination, methylation, Ac-Ub, acetylation–malonylation–ubiquitination, and acetylation–succinylation–ubiquitination with respect to co-occurring PTMs (Figure 5 and Supplementary Figure S7), we observed that all the plots depict phosphorylation to occur with maximum frequency around the motifs. For the singly identified modifications – acetylation, ubiquitination, and methylation – the second highest modification to be observed was of their own. Modifications in the same domains or linear stretches such as in p53 and p300 are well-known and reported to enhance their function (Yang and Seto, 2008). Serial modifications of multiple nearby sites may be required for a particular function. For example, acetylation in p53 occurs at six lysine residues (370, 372, 373, 381, 382, and 386) in the C-terminal region to activate transcription during DNA damage response. All these sites are also a target for ubiquitination, which results in cytoplasmic localization of p53 and proteosomal degradation. At site 382, methylation crosstalk with acetylation in nearby sites ensures acetylation of other sites in the vicinity. These types of simultaneous modifications of nearby residues were also observed at lysine positions 319, 320, and 321 in p53, where acetylation leads to stabilization, whereas ubiquitination leads to nuclear export and degradation (Yang and Seto, 2008). The regulation of protein function by simultaneous modifications of nearby sites represents an evolutionary control of metabolic processes that regulates the false activation of a process due to random PTM occurring at one site (Hunter, 2007; Venne et al., 2014).

### PTM Hotspots and Crosstalk Hotspot Regions in Proteins

**FIGURE 6:**
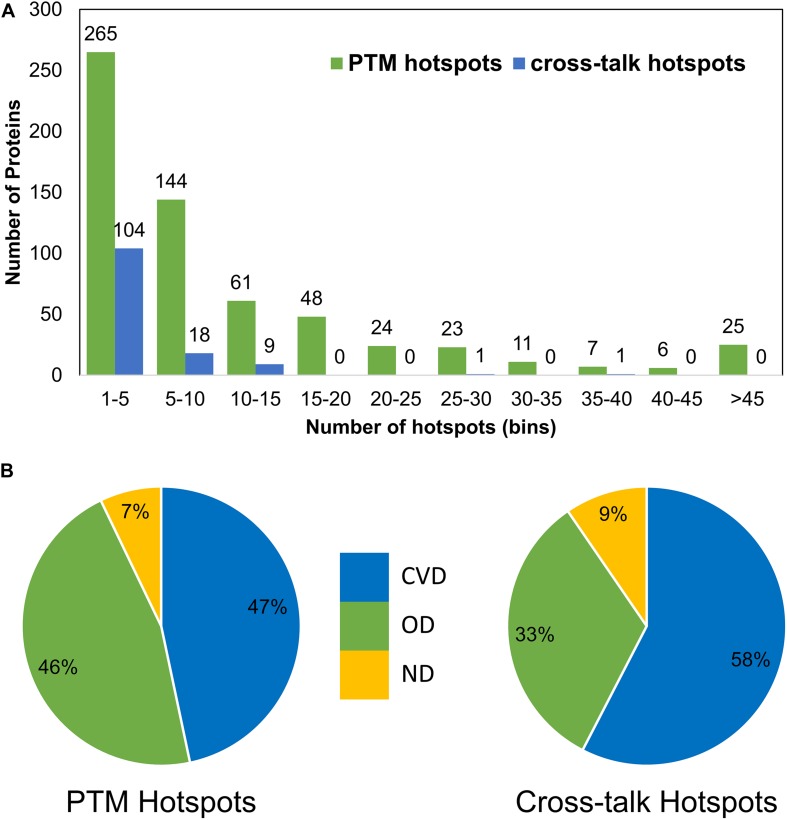
Post-translational modification and crosstalk hotspots in sirtuin interactors. **(A)** Frequency of proteins in each hotspot bin. **(B)** Disease category distribution in PTM hotspots (left) and crosstalk hotspots (right) shows their occurrence in CVDs and other diseases. While PTM hotspots have similar association with CVDs like OD, the crosstalk hotspots show much higher association (58%).

To identify the regions of PTM and crosstalk hotspots for their susceptibility to diseases, we divided the protein regions based on their PTM sites and crosstalk sites. We identified high-density stretches based on the number of PTM motifs containing more than five PTM sites or three crosstalk sites in one motif of 15 amino acids (aa) excluding the central modified site (Supplementary Table S2). We observed 614 such proteins that contain at least one stretch of 15 aa with more than five PTM sites and 133 proteins containing more than three crosstalk sites in at least one 15-aa stretch. Roughly half of the proteins in this list had ≥10 crosstalk or PTM hotspot sites (Figure 6A). We also observed that the distribution of PTM hotspots is comparable in OD (46%) and CVDs (47%), whereas the crosstalk hotspots in CVD proteins were comparatively much higher (58%) (Figure 6B). There were 112 proteins that contained both PTM hotspots and crosstalk hotspots. The 21 proteins that do not contain any PTM hotspot but had crosstalk hotspots were all involved in carbon and energy metabolism pathway (Supplementary Figure S8). We calculated sequence coverage for the proteins with PTM and crosstalk hotspots by stitching together all overlapping motifs of 15 aa for both categories of hotspots (Supplementary Table S2). We observed that histone proteins had the highest sequence coverage, closely followed by SRRM2. We then calculated a ratio of crosstalk hotspots to PTM hotspots and observed that the proteins with ratio ≥0.1 were involved in several cancers and aging-related diseases (Supplementary Figure S8), as most of the studies reflect phosphorylation-signaling aberrations in cancers.

### Variant Sites in PTM Motif

**FIGURE 7:**
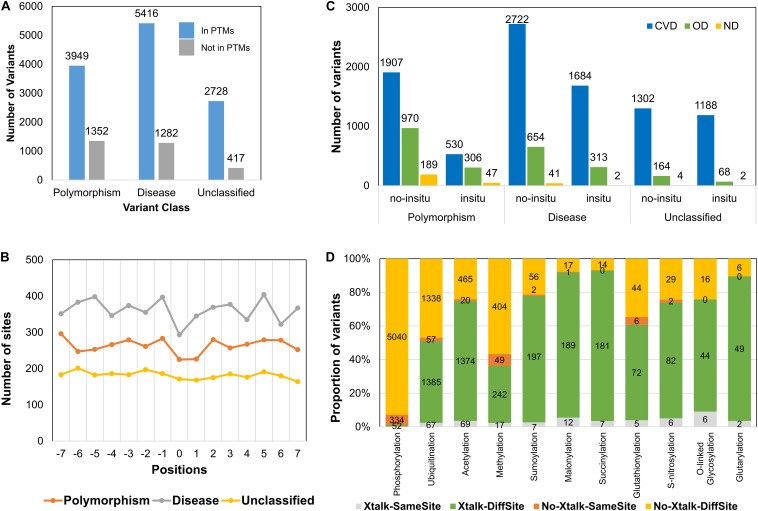
Distribution of variants in PTM motifs. **(A)** Number of motif-associated variants (MAVs) in each category (polymorphic, disease-related, or unclassified). **(B)** Motif-associated variants as observed in disease class of proteins and their occurrence in crosstalk motifs (*in situ*). **(C)** Number of MAVs observed at different positions in the PTM motif. **(D)** Proportion of MAVs in crosstalk/PTM motifs for each PTM type.

Genomic variations or single-nucleotide polymorphisms can change the protein sequence and thus function. Some of these variations can lead to change of amino acid harboring a modification and thus lead to functional aberration (Reimand et al., 2015). To investigate the presence of such aberrations in sirtuin interactor PTMs, we collated data from PTMvar (PSP) and Humsavar (UniProt). After redundancy removal, we collated information on 7,311 variants in sirtuin interactors. These variants mapped to ∼12,000 PTM sites in the ± 7-aa motif. Because any sequence change in the sites nearby a PTM can result in functional loss, we studied if these sites are already associated with any disease. Most of these variants present in PTM motif are known (5,416 variants) to cause diseases (Figure 7A). When we further divided these PTM motif-associated variants (MAVs) according to disease classification of the protein, we found that a higher number of variants fall in CVD-related proteins as compared to OD and ND (Figure 7B). Although the overall distribution of MAVs does not vary much for unclassified variants (unknown function) but for “disease-causing” MAVs, there is a dip observed at the site of modification (Figure 7C). These variants might lead to “loss-of-function” due to loss of modifications at the site and thus hamper the normal protein functioning. The dip is not highly pronounced, which suggests that the other nearby sites also have only slightly lesser but similar deleterious effect on functionality of PTM sites. Some of the changes might result in low functional capacity of the protein or disruption of PPIs, but others might lead to either a loss of function or the structure of a protein.

The effect of variants on protein structure and their presence in disordered regions is extensively investigated. Through disorder prediction, it has been observed that variants are more prominent in disordered regions as these have faster evolutionary rate as compared to ordered regions (Brown et al., 2002; Echave et al., 2016; Narasumani and Harrison, 2018). Unexpectedly, it was not found to be true in case of MAVs observed for sirtuin interactors (Supplementary Figure S9). This corroborated with our previous result on disorder and PTM sites (Figure 2C). We observed here that 67% of MAVs were observed in ordered, 17% in disordered non-binding, and 15% in disordered binding regions. In addition, when we divided MAVs according to the protein disease association, 77% of the MAVs were observed in CVD-associated proteins. This suggests that the MAVs may have a prominent role to play in connection to modification stability. To study the effect of MAVs on PTMs – whether at the site or away from it – we divided the MAV sites for different PTM types (Figure 7D). We observed that mostly lysine modifications, such as acylations, ubiquitination, sumoylation, and methylation, harbor MAVs in the crosstalk regions. This observation was expected as majority of modifications observed in these data occur on lysine residues. However, it was also surprising to see that majority of variants in phosphorylation sites fall in the PTM motif of non-crosstalk sites despite the presence of ∼300 phosphorylation and O-glycosylation crosstalks. We also observed cysteine modifications – glutathionylation and S-nitrosylation – both mapping a major part of their MAVs in crosstalk sites. Both of these modifications are known to regulate oxidative stress that might alter protein functions in different biological contexts (Beltran et al., 2000; Aparicio-Trejo et al., 2019; Valerio et al., 2019).

To understand the impact of MAVs on crosstalk with disease, we subdivided the sites observed in Figure 7D on the basis of disease classification of the proteins they were observed in (CVD, OD, or ND) (Supplementary Figure S10). While we did not observe any MAV for residues harboring crosstalk in ND, only ∼50 sites were observed in crosstalk motifs. Majority of MAVs were observed in crosstalk/no-crosstalk motifs in CVD category proteins but not at the site of PTM. It could be because the change of amino acid at the site of modification/crosstalk may not be well tolerated by cells and are probably lethal.

**FIGURE 8:**
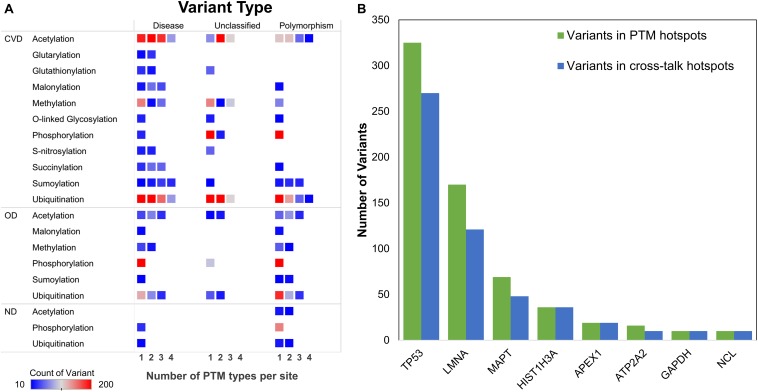
**(A)** Distribution of MAVs in disease-associated proteins with respect to the number of PTM types per site. **(B)** Proteins with ≥10 MAVs in PTM and crosstalk hotspot regions.

We further collated the MAVs observed in crosstalk sites and their known effects in various PTMs categorizing them according to the protein disease status (Figure 8A). We  observed that for the highest frequency of crosstalks (in acetylation and ubiquitination) both PTMs have a higher number of MAVs in crosstalk motifs of two, three, and four PTM types in CVD proteins and are known to co-occur with disease variants. However, this number goes down for both acetylation and ubiquitination in OD category for disease-related and simple polymorphisms. The highest number of MAVs was observed in phosphorylation motifs in CVDs (Supplementary Figure S11), but if subdivided based on PTM crosstalk, there are no sites observed in disease-related MAVs despite the presence of ∼300 crosstalk sites of O-glycosylation and phosphorylation (Figure 8A).

When the MAVs were studied for some PTMs, we observed that the pattern of MAVs in position specificity of PTM motif shows an opposite pattern for acetylation and ubiquitination in CVD and OD (Supplementary Figure S12). For phosphorylation, it was more or less similar for CVD- and OD-related proteins. This suggests that the interplay of variants in acetylation and ubiquitination sites might lead to loss or gain of protein function leading to disruption of cellular homeostasis (Karve and Cheema, 2011).

We also mapped the MAVs and their co-occurrence in the PTM hotspot regions and crosstalk hotspot regions. TP53 emerged to be the highest number of variant containing protein in PTM and crosstalk hotspots closely followed by LMNA and MAPT (Figure 8B). LMNA was also observed to contain 14 different PTM types on 240 PTM sites. LMNA is a component of nuclear lamina and is important for nuclear assembly, chromosomal organization, and cardiac homeostasis. Other proteins enriched in both categories of hotspots and mapping with variants included MAPT, ATP2A2, NCL, APEX1, and HIST1H3A. A GO analysis reveals that all the above proteins are involved in regulation of metabolic process, response to oxidative stress, and chromosomal reorganization (Supplementary Figure S13).

## Discussion

Sirtuins are an important class of deacetylases implicated in many metabolic diseases, such as cancer, diabetes, and CVDs, as well as Alzheimer disease and aging (Yamamoto et al., 2007; Masri, 2015; Grabowska et al., 2017; O’Callaghan and Vassilopoulos, 2017; Costa-Machado and Fernandez-Marcos, 2019; Gomes et al., 2019). These deacetylases are the central players controlling the metabolism. Any deregulation in these proteins or their interactors can lead to disease. PTMs are a hidden regulatory layer that can change a protein’s function, localization, or interactions at a rapid timescale – either alone or through crosstalk with other PTMs (Walsh et al., 2005; Karve and Cheema, 2011; Lin and Begley, 2011). Metabolic changes are brought about by multiple PTM interactors of sirtuins by controlling the regulation of proteins involved in transcription and carbon metabolism (Soskic et al., 2008; Sheeran and Pepe, 2016). Despite the known regulation of PTMs in sirtuins, the effect of PTMs on their interactors has not been studied. While targeting sirtuins directly using small molecules or other approaches might be more challenging because of their central importance and can cause adverse effects, targeting the interactors for specific disease outcomes is a tantalizing proposition. This requires deeper understanding of the interactors with respect to their PTMs and their crosstalks. However, it is challenging because of several reasons. First, not all PTM sites are functionally important. Second, establishing a causative link between PTM and diseases is challenging itself. Third, phenotype is an outcome of complex interactions of multitude of PTMs, each contributing to the outcome without having a one-to-one correspondence. Thus, a direct biological study of PTM and their crosstalks is going to be highly challenging and unwieldy at scale. An association study using big data from biological database using bioinformatics analysis is an efficient method to study such relationships, which may be probed deeper by selectively picking targets.

In this study, we have studied the PTMs and their crosstalks in SIRTs and their interactors carefully selected from several databases. These data are a compendium of curated resources for studying how PTMs are associated in disease, structural disorder, crosstalks, and variants. We show that sirtuin interactors (1,258) have higher PTM density (83 sites and 5 types) than the complete human proteome (4.5 sites and 2 types). The PTM distribution is surprisingly found to be in ordered regions (∼60–70%). Because the sirtuin interactors are the central players in metabolism, they may carry a different character. This suggests that despite their moonlighting functions, these proteins are evolutionarily stable, and their multiple interactions are not prone to rewiring or transient in selection process. This also suggests that their functions are more critical than the average human protein due to which they also show a higher proportion of disease-associated proteins (544 in CVD and 574 in OD).

We observed about 11,000 competitive crosstalk sites in 1,046 proteins (∼83% of sirtuin interactors) with nearly half of the sites representing the competition of acetylation and ubiquitination. Most of the histones contained extensive crosstalks up to seven PTMs competing for the same site. While most of the sirtuin interactors contained at least one crosstalk site, there were 367 proteins harboring ≥10 crosstalk sites, with AHNAK protein containing 339 PTM crosstalk sites. AHNAK is a calcium transporter known to be involved in cancers and CVDs. We studied how PTM crosstalks are distributed in the PTM motifs along with disease-related variants. We observed that most functionally validated disease causal variants occur at the PTM/crosstalk site or within the motif (5,416 variants). Their association in CVDs is much higher (∼77%), denoting that such proteins are viable targets for further studies for their roles in CVDs and metabolic disorders. We also defined *PTM* and *crosstalk hotspots* (≥5 and ≥3, respectively) as regions containing higher densities that are driving major metabolic pathways. The PTM crosstalks are enriched in carbon metabolism, glycolysis, pyruvate and propanoate metabolism, gluconeogenesis, fatty acid degradation, and amino acid biosynthesis and degradation pathways (Supplementary Figure S8). On the other hand, the proteins containing both types of hotspots and higher fraction of crosstalk sites are important players in cancers, aging, apoptosis, and other diseases (Supplementary Figure S8).

These hotspots were also found to be closely associated with disease-related variants in crosstalk sites for acetylation and ubiquitination crosstalk (Figure 8A and Supplementary Figure S12). This crosstalk is well-known, but its importance in sirtuin interactors and metabolism is understudied. We also highlighted presence of other types of *in situ* crosstalks such as sumoylation–ubiquitination, acylation–ubiquitination, glutathionylation–S-nitrosylation, and O-glycosylation–phosphorylation and their regulation of protein function through disruption of PTM site or motif. When we analyzed the presence of these variants in PTM and crosstalk hotspots, we observed seven proteins having ≥10 variants sites in both hotspots. Although the majority of these sites were observed in TP53, studied extensively in metabolic diseases, we also observed LMNA, which is known to maintain cardiac homeostasis. Similarly, other proteins also perform important functions such as regulating metabolic processes and response to oxidative stress. Further mechanistic studies on these proteins can uncover their interesting roles in CVDs and have potential for therapeutic applications. These data can help in generating exploratory hypothesis for mechanistic insights into protein function using PTMs and crosstalk hotspots. The functionally validated sites can potentially be useful for newer leads into disease mechanisms, biomarkers, or as drug targets.

## Data Availability Statement

The raw data supporting the conclusions of this article will be made available by the authors, without undue reservation, to any qualified researcher.

## Author Contributions

SA and AY contributed to design of the study. SB and AY classified diseases. SA and AY analyzed data with inputs from NT and SB. SA wrote the first draft. NT, SB, SA, and AY contributed to writing and editing the manuscript.

## Conflict of Interest

The authors declare that the research was conducted in the absence of any commercial or financial relationships that could be construed as a potential conflict of interest.

## Footnotes

1 http://biomine.cs.vcu.edu/servers/fMoRFpred/

2 http://www.pantherdb.org/geneListAnalysis.do

3 https://string-db.org/

4 https://ptmcode.embl.de/data.cgi

5 https://www.uniprot.org/docs/humsavar

6 https://www.phosphosite.org/staticDownloads
