# Supplementary material for: Post-translational Modification Crosstalk and Hotspots in Sirtuin Interactors Implicated in Cardiovascular Diseases
Source: Front Genet. 2020 Apr 30;11:356. doi: 10.3389/fgene.2020.00356 (PMC7204943; doi:10.3389/fgene.2020.00356)
Supplement: Supplementary file 1 [file Presentation_1.pdf]

Supplementary Material

1 Supplementary Figures

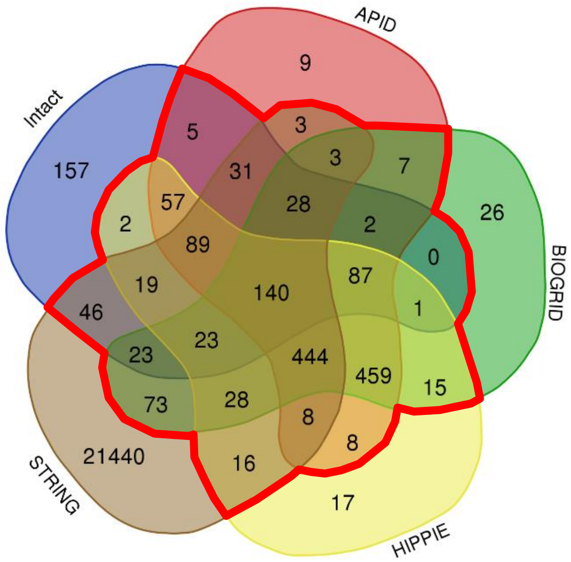

Supplementary Figure 1: The sirtuins interactors from five interaction databases with their overlaps (inside red outline). Proteins common to two PPI databases were selected for further analysis.

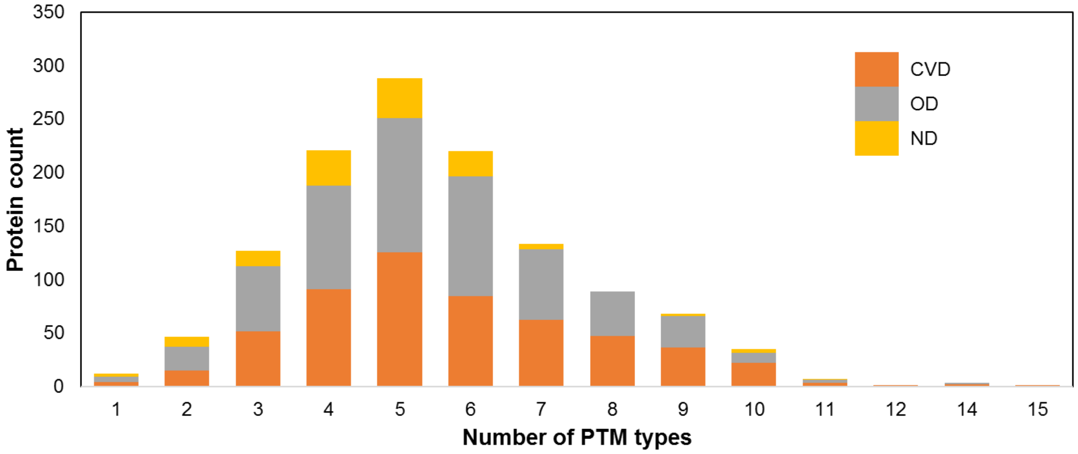

Supplementary Figure 2: The proteins with a particular number of PTM types and disease category are shown. Most proteins are either in CVDs or OD while ND proportion is low. CVDs are associated with approximately half of the proteins with other major proportion mapping to other diseases, being a conglomeration of several disease types.

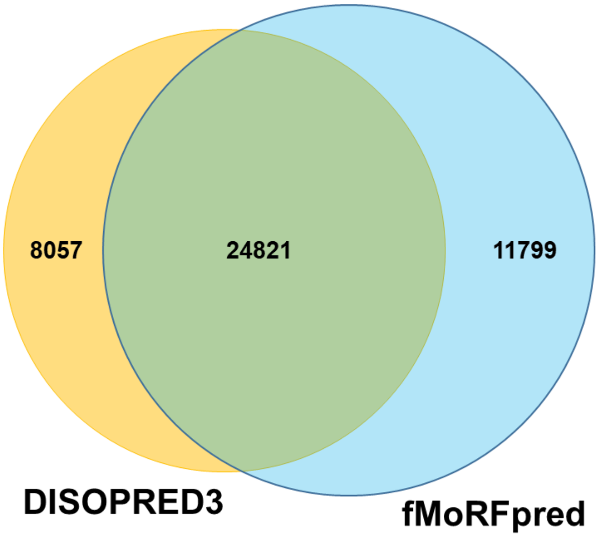

Supplementary Figure 3: Comparison of PTM sites in disordered regions as predicted by DISOPRED3 and fMoRFpred software. Although there was less agreement (Figure 2C) between the disordered binding and non-binding sites, the overall disorder had reasonably good agreement.

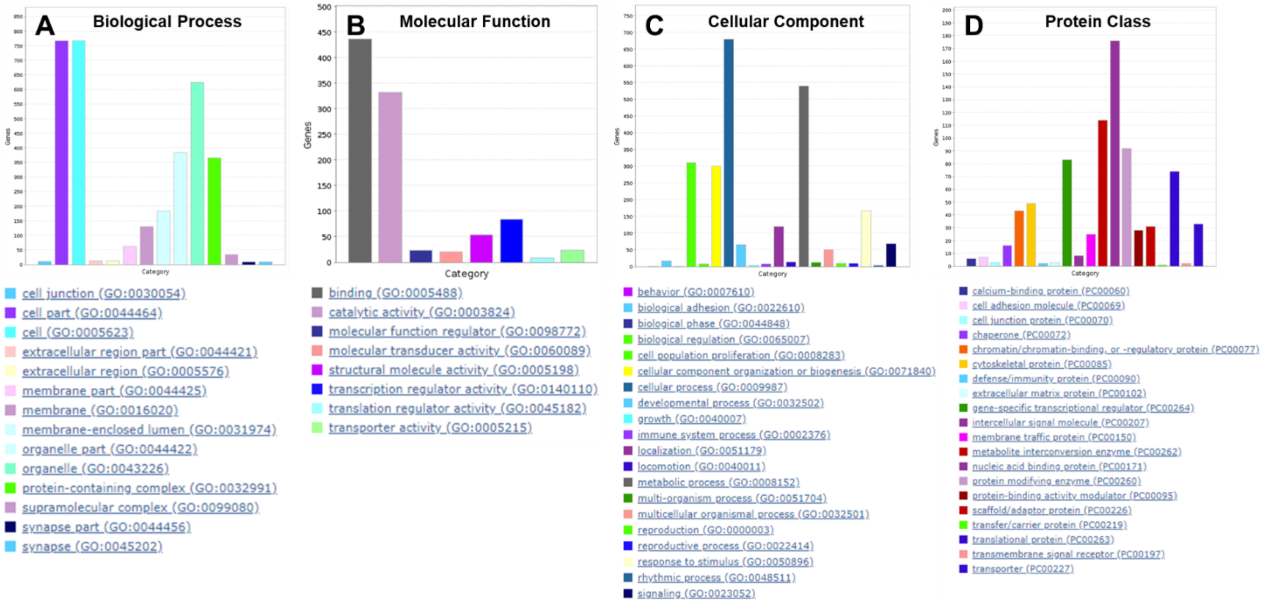

Supplementary Figure 4: Gene Ontology (GO) analysis for the SIRT interactors- (A) Biological Process, (B) Molecular Function, (C) Cellular Component, and (D) PANTHER Protein Class. The interactors majorly control the metabolic processes in diverse biological protein classes.

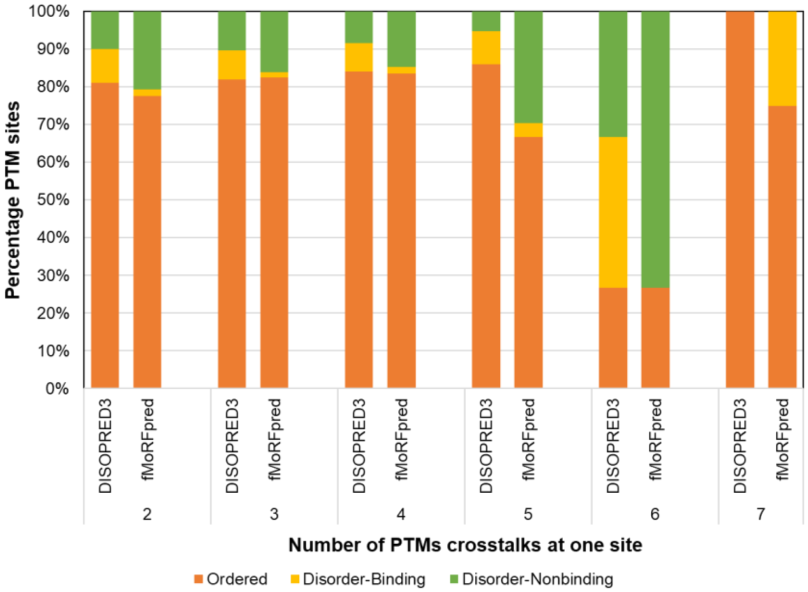

Supplementary Figure 5: Percentage of sites across structural regions for different frequency of PTM crosstalks at a site for DISOPRED3 and fMoRFpred prediction tools.

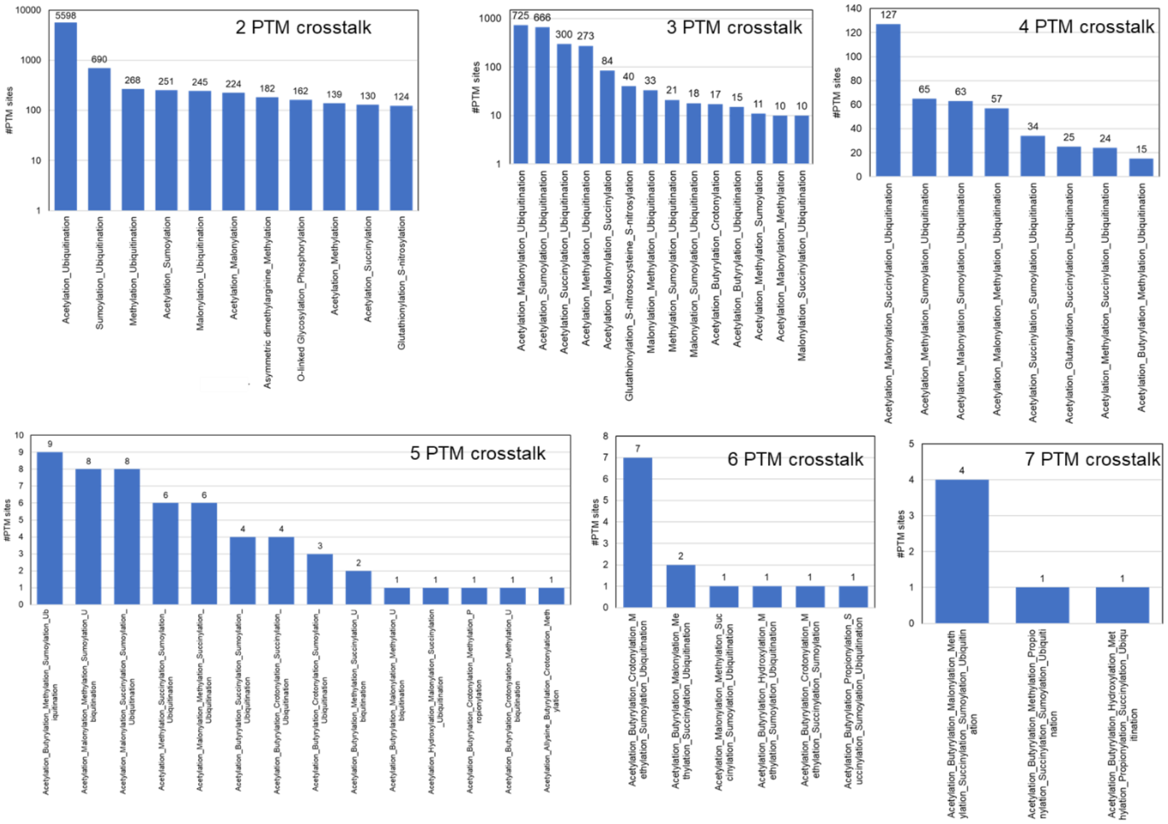

Supplementary Figure 6: Number of Sites observed for each type of PTM crosstalk (in-situ) in sirtuin interactors. For 2 and 3 PTM crosstalk data, PTM pairs with less than 100 sites were removed. For 4 PTM crosstalk data, pairs with less than 15 sites was removed.

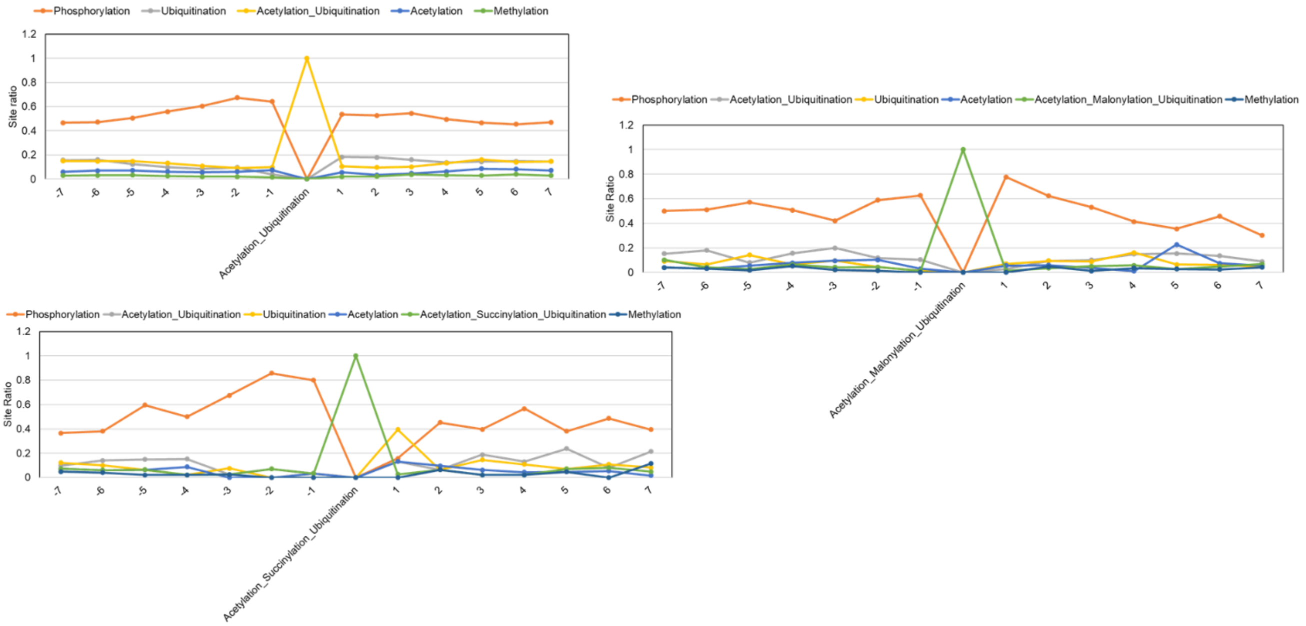

Supplementary Figure 7: Occurrence of PTMs in the motif of crosstalk pairs: acetylation-ubiquitination, acetylation-malonylation-ubiquitination and acetylation-succinylation-ubiquitination.

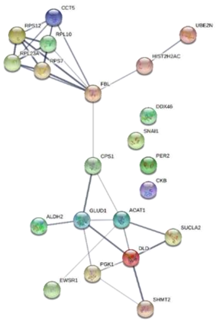

KEGG Pathways in Crosstalk only	Proteins with >10 % of cross-talk/ PTM hotspots
Carbon metabolism	Systemic lupus erythematosus
Metabolic pathways	Alcoholism
Glyoxylate and dicarboxylate metabolism	Melanoma
Propanoate metabolism	Non-small cell lung cancer
Pyruvate metabolism	Transcriptional misregulation in cancer
Ribosome	Proteoglycans in cancer
Valine, leucine and isoleucine degradation	Pancreatic cancer
Glycolysis / Gluconeogenesis	Chronic myeloid leukemia
Biosynthesis of amino acids	Acute myeloid leukemia
Nitrogen metabolism	Longevity regulating pathway
Arginine biosynthesis	Small cell lung cancer
Citrate cycle (TCA cycle)	Prostate cancer
Alanine, aspartate and glutamate metabolism	Toxoplasmosis
Glycine, serine and threonine metabolism	Sphingolipid signaling pathway
Tryptophan metabolism	Neurotrophin signaling pathway
Fatty acid degradation	Apoptosis

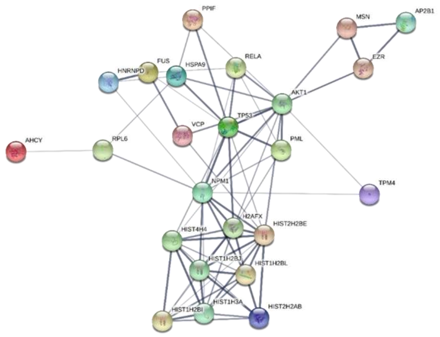

Supplementary Figure 8: Interaction network of proteins harboring only crosstalk hotspots (21 proteins) (left panel) and proteins containing both PTM and crosstalk hotspots (24 proteins) (right panel). The table shows the KEGG pathways enriched for both the lists respectively.

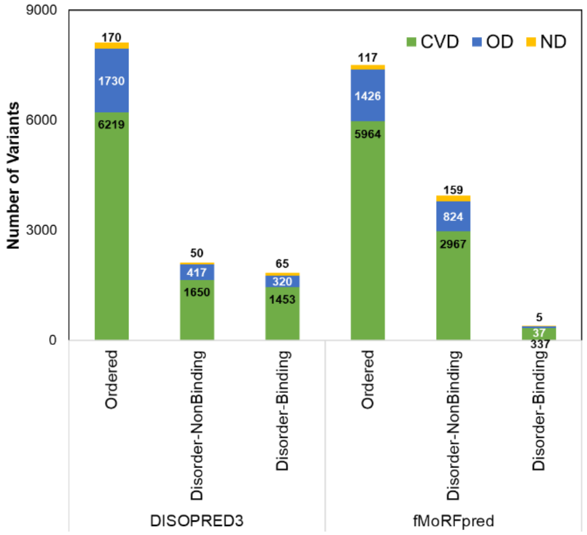

Supplementary Figure 9: Distribution of motif associated variants (MAVs) in the structural region of a protein as predicted by DISOPRED3 and fMoRFpred. The MAVs are further divided based on the disease association of the proteins. Most MAVs are observed in CVD associated proteins.

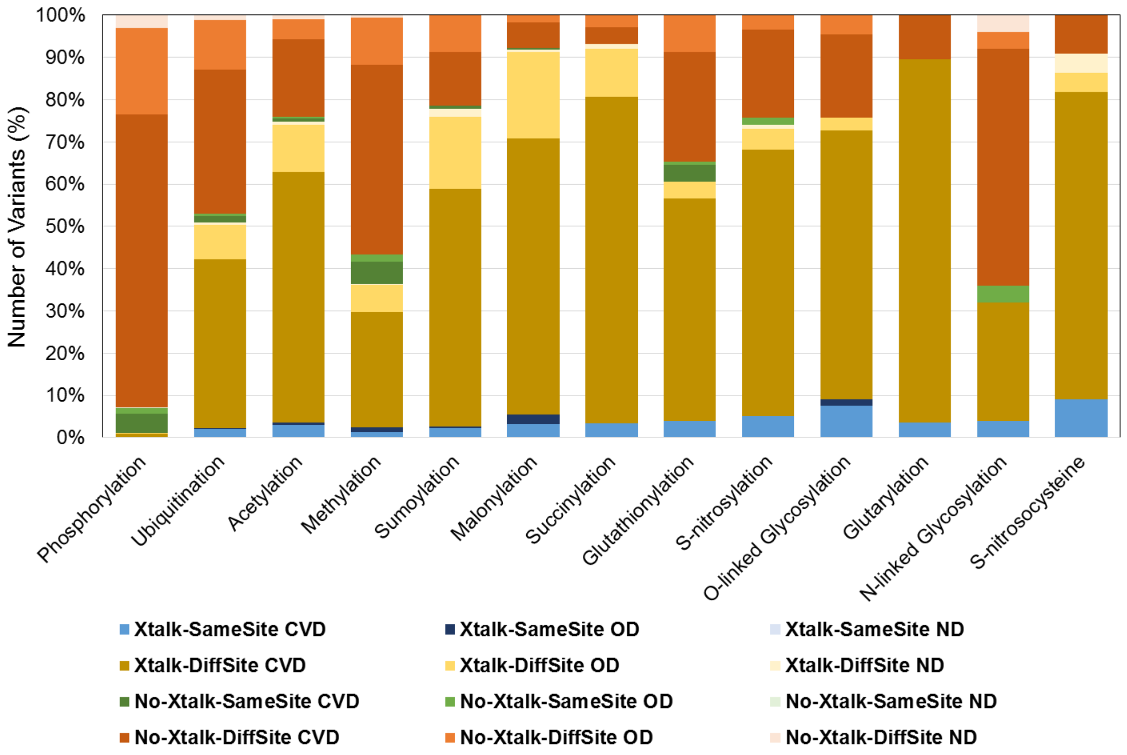

Supplementary Figure 10: Number of variants in site of PTM/crosstalk or in the motif with respect to its presence in diseases-associated proteins. Most of the variants were observed in motif of CVD proteins.

		Disease	Polymorphism	Unclassified
CVD	Phosphorylation	1,877	1,326	854
	Ubiquitination	986	536	678
	Acetylation	769	294	510
	Methylation	225	80	258
	Succinylation	134	25	12
	Malonylation	121	36	7
	Sumoylation	90	69	29
	Glutarylation	50	2	5
	Glutathionylation	45	10	55
	S-nitrosylation	43	12	51
	O-linked Glycosylation	25	15	20
	N-linked Glycosylation	11	11	0
	S-nitrosocysteine	10	6	4
	Hydroxylation	6	8	0
ND	Phosphorylation	27	148	4
	Ubiquitination	13	35	1
OD	Phosphorylation	432	670	95
	Ubiquitination	229	299	70
	Acetylation	136	148	37
	Methylation	64	69	9
	Sumoylation	28	32	9
	Malonylation	24	25	5
	Succinylation	20	6	3
	Glutathionylation	9	6	2
	S-nitrosylation	8	4	0
	Asymmetric dimethylarginine	8	4	0

Measure Values: 5.0 – 1,000.0

Supplementary Figure 11: PTM-type wise site count in different classification of variants (disease-related, polymorphic or unclassified) and disease associated proteins (CVD, OD and ND). Mostly the MAV sites were observed in CVD related proteins.

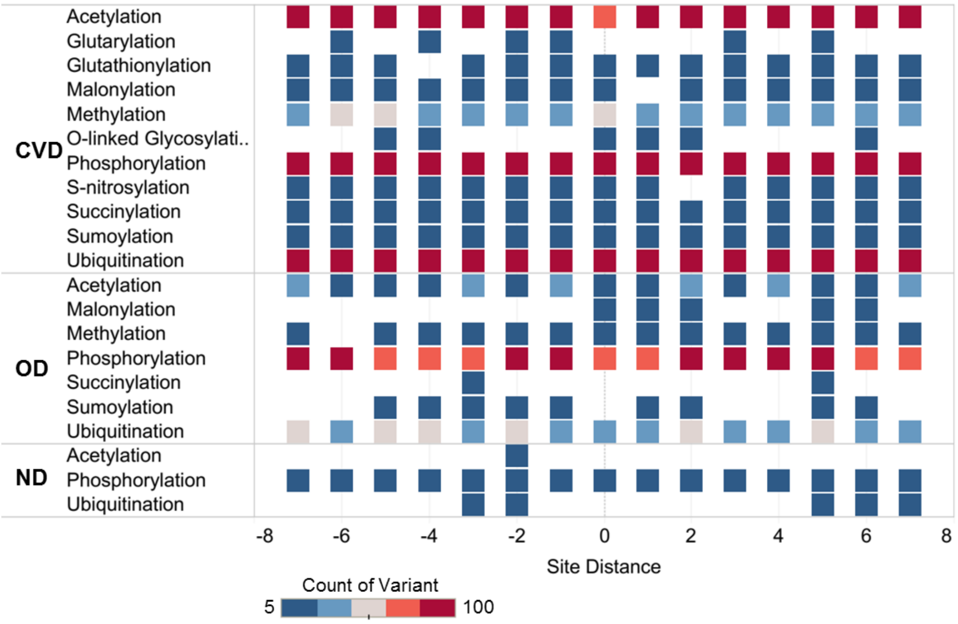

Supplementary Figure 12: Distribution of MAVs in specific PTM motifs.

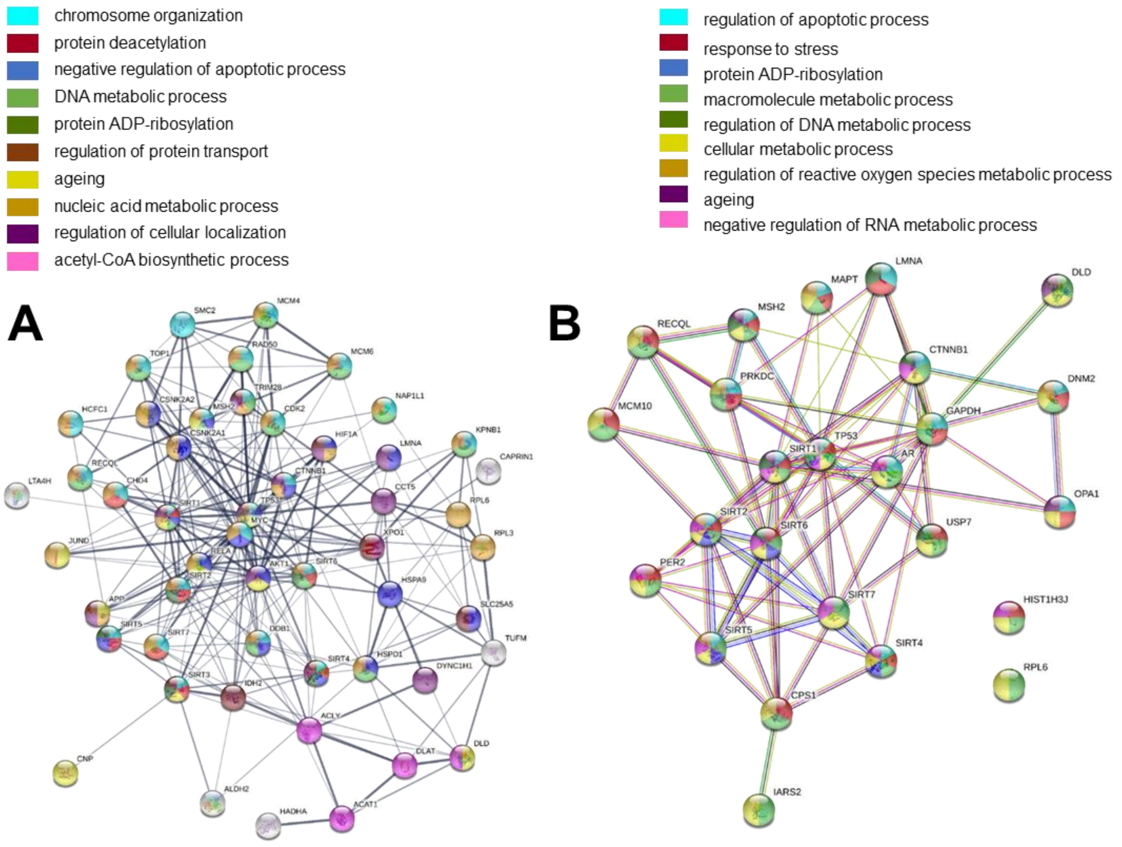

Supplementary Figure 13: Interaction networks and pathway enrichment of proteins containing variants in crosstalk motifs and interact with multiple SIRTs. (A) Proteins (44) containing variants in the motif of crosstalk sites. (B) Proteins (19) containing variant at the site of crosstalk leading to loss of PTM residue.
